# The gut microbiome in oral health and disease: evidence toward bidirectional oral-gut axis communication

**DOI:** 10.3389/fmicb.2026.1817689

**Published:** 2026-06-09

**Authors:** Vikram Khanna, Smita Kumar, Sumit Kumar, Saurabh Verma, Anastasios Grigoriadis, Abhishek Kumar

**Affiliations:** 1 Department of Oral Medicine and Radiology, Faculty of Dental Sciences, King George's Medical University, Lucknow, Uttar Pradesh, India; 2 Program Science-Based Research at the University of Manitoba, Winnipeg, MB, Canada; 3 Indian Health Action Trust, Lucknow, Uttar Pradesh, India; 4 King George's Medical University, Lucknow, India; 5 Multi-Disciplinary Unit-Department of Health Research, King George's Medical University, Lucknow, Uttar Pradesh, India; 6 Humanex Technologies Solutions, Abu Dhabi, United Arab Emirates; 7 Division of Oral Rehabilitation, Department of Dental Medicine, Karolinska Institutet, Stockholm, Sweden; 8 Academic Center for Geriatric Dentistry, Stockholm, Sweden; 9 Department of Conservative Dentistry and Endodontics, Dr. D. Y. Patil Dental College and Hospital, Dr. D. Y. Patil Vidyapeeth (Deemed to be University), Pimpri, Pune, India

**Keywords:** bidirectional oral gut axis, oral dysbiosis, gut dysbiosis, oral diseases, gut-oral axis, oral flora

## Abstract

The oral-gut microbiome axis has largely been seen as a unidirectional framework, in which dysbiotic oral flora is considered to contribute to gastrointestinal and systemic disease. However, recent evidence now challenges this view, indicating that gut microbial imbalance can act upstream to modulate oral immune homeostasis and disease susceptibility. Therefore, in the current perspective paper, we present a structured narrative review that synthesizes recent evidence from human microbiome, immunological, and genetic studies to propose a hypothetical mechanistic model in which gut dysbiosis may contribute to oral pathology. The literature discussed was identified through a targeted keyword-based search of major databases and complemented by manual screening of reference lists to capture relevant studies. Analyzing the evidence from human case-control and longitudinal cohort studies, as well as Mendelian randomization analysis, we identify convergent pathways linking gut dysbiosis to oral disease. These include systemic immune priming in autoimmune disorders with oral manifestations, depletion of gut-derived metabolites, such as short-chain fatty acids, that regulate epithelial barrier function and inflammation, and dysbiosis-associated barrier disruption that facilitates the systemic dissemination of microbial products and inflammatory mediators. Through these mechanisms, gut microbial imbalance contributes to chronic inflammatory conditions, altering host response and susceptibility to dental and mucosal diseases. In contrast, studies in healthy individuals show minimal oral-gut microbial overlap, supporting a model in which physiological compartmentalization is maintained in health and disrupted primarily under dysbiotic conditions. This synthesis reframes oral disease as host–microbiome dysregulation, highlighting gut microbiota as a driver of oral immune pathology.

## Introduction

The gut and oral cavity are the two main reservoirs of microbes in the body, comprising trillions of bacteria. The current evidence suggests that dysbiosis in these ecosystems plays a significant role in the development of various systemic conditions (Kunath et al., 2024). The gut plays a mediating role in how the body's organs communicate. Through its connections with the brain, liver, skin, and lungs, it can influence inflammation throughout the body, affect cognitive function, contribute to autoimmune disorders, and cause nutritional imbalances, thereby impacting an individual's overall health. Similarly, the oral cavity is part of several interconnected pathways. Recent reviews have acknowledged the role of the oral-gut microbiome axis in health and disease (Kunath et al., 2024; Peng et al., 2022). Therefore, in this review, we present a brief overview of the evolving dynamics between the oral and gut microbiomes, with emphasis on emerging evidence supporting bidirectional communication and its relevance to oral and systemic health, based on recent literature.

## The oral and gut microbiomes: shared principles, distinct ecosystems

The connection between oral and gut microbiota is generally studied in terms of how the oral cavity influences the gut environment. The complex pathways through which oral microbiota or diseases affect the gut include enteral transmission (Park et al., 2021), hematogenous translocation of bacteria and their toxins (Yamazaki and Kamada, 2024), immune responses and inflammation (Zhong et al., 2024), and metabolic disturbances (Xu et al., 2025). Poor oral hygiene, personal deleterious habits like smoking, improper diet, and diseases like periodontitis, obesity, and diabetes have all been identified in the literature as contributing factors (Lertpimonchai et al., 2017). These factors can lead to pathobiont dysbiosis and translocation, resulting in various gut pathologies, including inflammatory bowel disease, celiac disease, and Crohn's disease, which in turn impact systemic health and quality of life (Yamazaki and Kamada, 2024; Rajasekaran et al., 2024; Zhao et al., 2025). Conversely, bacteria and their metabolites originating in the gut can influence distant oral sites. A healthy gut supports the oral cavity with nutritional support, stronger immunity, and reduced inflammation (Zhao et al., 2025). Independent studies have linked gut dysbiosis to oral diseases, such as periodontitis (Miyauchi et al., 2025; Yamazaki, 2023), aphthous ulcer (Jin et al., 2024; Wang et al., 2022), and Sjogren's syndrome (Yao et al., 2022; Mandl et al., 2017). Therefore, it is suggested that the oral cavity acts as a gateway for microbial colonization, shaping the composition of subsequent microbial communities throughout the body. While the link between oral health and the gut is well-established, the causal relationships and underlying mechanisms, such as systemic inflammation or altered metabolite production, are currently active areas of investigation. A comprehensive compilation of studies demonstrating gut dysbiosis affecting the oral cavity is the first step toward understanding the underlying pathways. Although bidirectional interactions between the oral cavity and the gastrointestinal tract have been proposed, the evidence supporting these directions is not equivalent. The oral-to-gut pathway is relatively well described, with studies demonstrating the translocation of oral microbes to the gastrointestinal tract and their potential role in systemic inflammation and disease (Kageyama et al., 2023). In contrast, the reverse direction, i.e., how gut microbiota may influence oral conditions, remains far less clearly established. Proposed mechanisms include systemic immune modulation, circulating microbial metabolites, and inflammation originating from gut dysbiosis that may affect oral tissues. However, direct mechanistic and clinical evidence linking alterations in gut microbial ecology to specific oral manifestations remains limited, and the oral consequences of gut dysbiosis have not yet been systematically characterized in the literature.

Understanding gut dysbiosis and its impact on oral health is crucial in clinical dentistry, as it demonstrates how systemic factors can influence periodontal disease and oral microbiota beyond local oral conditions. For example, long-term use of systemic antibiotics can induce gut dysbiosis, worsening periodontitis by disrupting oral microbial balance and immune regulation, underscoring the need for regular periodontal assessment during such treatment (Yuan et al., 2023). A structured, keyword-based literature search was conducted to address the research question: “How does gut dysbiosis and gut microbial composition influence the oral microbiota and oral diseases?” The search was performed across three major electronic databases, PubMed, Embase, and Web of Science, to capture relevant experimental, clinical, and observational studies. The primary aim of this short review was to synthesize and critically appraise current evidence regarding the role of gut dysbiosis in shaping oral microbial dysbiosis and its potential implications for oral health and disease. We attempted to identify all recent (5-year) studies published in English, in peer-reviewed journals, and in human subjects that indicate an association between gut dysbiosis and oral flora. The search strategy used the key words:(((((((((oral gut axis) OR (oral gut link)) OR (Microbial translocation)) OR (interplay)) OR (dysbiosis)) OR (connection)) OR (crosstalk)) OR (bidirectional relationship)) AND (((((((gut microbiome) OR (gut microbiota)) OR (gut flora)) OR (gastrointestinal microbiome)) OR (intestinal microbiome)) OR (faecal microbiome)) OR (bowel flora))) AND (((((((Oral microbiome) OR (oral microbiota)) OR (oral flora)) OR (mouth bacteria)) OR (salivary microbiome)) OR (dental plaque)) OR (periodontal pathogens)). The articles were screened based on their titles and abstracts for relevance to the research question. The full text of the screened articles was downloaded. In total, we identified 19 articles relevant to our research question after applying the inclusion and exclusion criteria, as summarized in Table 1.

**Table 1:** Characteristics of the studies.

Study no.	References	Year	Type of study	Investigative tests	Oral condition	Result
1	Wang et al. (2024)	2024	Mendelian randomization study	Genome-wide association study data for gut microbiota	Sjogren's Syndrome	*Eubacterium coprostanoligenes* group mediated its protective effect by reducing CXCL6 levels in SS
2	Li et al. (2022)	2022	Case control study	16S Ribosomal RNA analysis of the gut microbes	Primary Sjogren's Syndrome (pSS)	*Bacteroides, Megamonas*, and *Veillonella* were significantly more abundant in pSS patients and positively correlated with their clinical indicators.
3	Yang et al. (2022)	2022	Case control study	16S rRNA gene amplification in pSS and healthy	Primary Sjogren's Syndrome (pSS)	pSS gut microbiota is characterized by increased abundances of proinflammatory microbes, especially *Escherichia-Shigella*, and decreased abundances of anti-inflammatory microbes
4	Mendez et al. (2020)	2020	Case control study	16S-rRNA- gene sequencing	Primary Sjogren's Syndrome (pSS)	Subjects with Dry eye had depletion of *Firmicutes* and an expansion of *Proteobacteria, Actinobacteria, and Bacteroidetes* compared to controls.
5	Kim et al. (2021)	2021	Case control study	16S-rRNA- gene sequencing	Behcet's disease and Recurrent Aphthous stomatitis (RAU)	Active BD patients had a significantly higher *fecal Bacteroides uniformis* than their matched HCs and patients with the disease in an inactive state. The salivary *Rothia mucilaginosa* group was higher in BD patients than in RAU patients.
6	Liu et al. (2025)	2025	Case control study	Lactulose hydrogen methane breath testing small intestine bacterial overgrowth (SIBO)	RAU	RAU patients are at a higher risk of anxiety and gut microbiota dysbiosis, which could potentially escalate the severity of RAU
7	Jin et al. (2024)	2024	Mendelian randomization study	Genome-wide association study data for gut microbiota	Oral ulcers	Three gut microbiota taxa were positively associated with mouth ulcers: *Holdemania, Oxalobacter, and Ruminococcaceae* UCG011, while four gut microbiota taxa were negatively associated with mouth ulcers: *Actinobacteria, Lactobacillales, Oscillospira, and Phascolarctobacterium*.
8	Wang et al. (2025)	2025	Mendelian randomization study	Genome-wide association study data for gut microbiota	Dental caries	*Eubacterium-brachy* group and *Terrisporobacter* has a positive impact on the progression of dental caries, while *Escherichia, Shigella, Oscillibacter, Ruminococcaceae* UCG014, *and Oscillospira* hurt caries development.
9	Zheng et al. (2023)	2023	Epidemiological study	16S Ribosomal RNA analysis of the gut and oral microbes	Dental Caries	The caries group showed greater richness in plaque samples and fecal samples.
10	Ye et al. (2023)	2023	Mendelian randomization study	Genome-wide association study data for gut microbiota	Periodontitis	Order *Enterobacteriales*, family *Bacteroidales* S24.7group, genus *Lachnospiraceae* UCG008, genus *Prevotella* 7, and order *Pasteurellales* may be associated with a higher risk of periodontitis, while genus *Ruminiclostridium* 6 may be linked to a lower risk.
11	Li et al. (2021)	2021	Case control study	16S-rRNA-gene sequencing gut microbes	Periodontitis	Butyrate-producing bacteria were decreased in the gut microbiota of the periodontitis group, including *Lachnospiraceae NK4A136 group, Eubacterium fissicatena group, Eubacterium coprostanoligenes group*, and *Ruminococcaceae UCG-014*, which were negatively correlated with serum HbA1c
12	Amado et al. (2020)	2020	Case control study	16S-rRNA-gene sequencing gut microbes and oral microbes	Periodontitis	Grade C/Molar incisor periodontitis presented a higher abundance of sulfidogenic bacteria in the feces, such as *Desulfovibrio fairfieldensis, Erysipelothrix tonsillarum and Peptostreptococcus anaerobius* were found in higher concentrations than in controls.
13	Lin et al. (2025)	2025	Mendelian randomization study	Genome-wide association study data for gut microbiota	Gingivitis	*Negativicutes, Verrucomicrobiae*, genus *Butyricicoccus, Eubacterium, Lactobacillus*, order *Selenomonadales*, and *Verrucomicrobiales* were associated with a higher risk of acute gingivitis. In contrast, family *Peptostreptococcaceae*, genus *Coprococcus*2, and genus *Lachnospiraceae* UCG001 were linked to a lower risk of acute gingivitis. Class *Erysipelotrichia, Methanobacteria, Verrucomicrobiae*, family *Defluviitaleaceae, Erysipelotrichaceae, Methanobacteriaceae, Verrucomicrobiaceae*, genus *Akkermansia, Christensenellaceae* R 7group, *Defluviitaleaceae* UCG011, *Methanobrevibacter*, genus *Paraprevotella, Senegalimassilia*, order *Erysipelotrichales, Methanobacteriales, Verrucomicrobiales*, and phylum *Cyanobacteria* were linked to a higher risk of chronic gingivitis, while family *Clostridiales* vadin BB60 group, genus *Allisonella, Dorea*, and *Lachnospiraceae* UCG004 were linked to a lower risk of chronic gingivitis.
14	Xu et al. (2024)	2024	Mendelian randomization study	Genome-wide association study data for gut microbiota	Periodontitis and Bleeding gingivitis	*Eubacterium xylanophilum* and *Lachnoclostridium* were associated with a reduced risk of gum bleeding, whereas *Anaerotruncus, Eisenbergiella*, and *Phascolarctobacterium* were linked to a reduced risk of periodontitis. Conversely, *Fusicatenibacter* was associated with an elevated risk of Periodontitis.
15	Zilberstein et al. (2025)	2025	Observational study	16S-rRNA-gene sequencing gut microbes and oral microbes	Periodontitis	Active Inflammatory Bowel Disease is associated with severe periodontal disorders and higher relative abundances of putative ‘pro-inflammatory' microbiota in the oral cavity.
16	Bartha et al. (2025)	2024	Longitudinal Study	16S-rRNA-gene sequencing	Ulcerative mucositis after allogenic stem cell transplantation	An increased abundance of *Enterococcus* species in the ulcerative and non-ulcerative groups post-transplantation.
17	Chen et al. (2024)	2024	Observational study in Oral cancer treatment patients	16S-rRNA- gene sequencing gut microbes	Oral Mucositis	*Bacteroidetes* showed an upward trend while *Proteobacteria* declined in higher grades of acute mucositis. Low-abundant Proteobacteria were significantly correlated with high-grade acute oral mucositis. *Lactobacillales* and *Actinomycetales* were specifically found in the group with better life quality. However, *Clostridia_UCG_014, Eubacteriaceae, UCG_010*, and *Moraxellaceae* were uniquely abundant in the worst life quality.
18	Matsui et al. (2024)	2024	Comparative study	Oral and fecal microbes' detection	Oral Squamous Cell Carcinoma	*Porphyromonas* and *Prevotella* were significantly more abundant in patients with OSCC than in HCs^*^
19	Nishimoto et al. (2023)	2023	Case control study	16S-rRNA-gene sequencing in oral and gut microbes	Oral microbiome and metabolome	Minimal oral gut axis in healthy and older individuals having more than 26 teeth.

^*^HCs, Healthy controls.

## Bidirectional oral–gut axis: immune, metabolic, and microbial interaction

**Figure 1:**
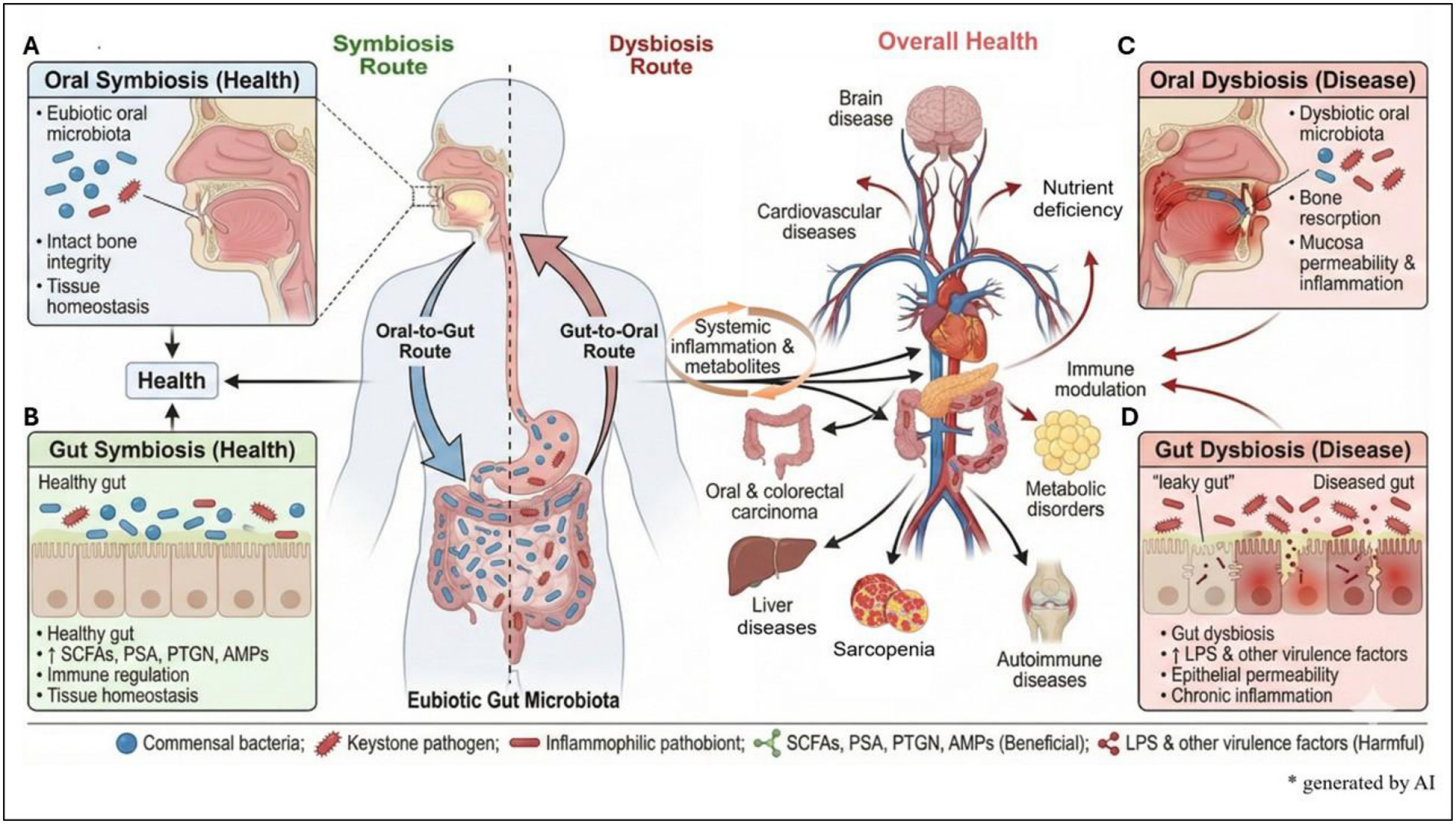
The bidirectional mechanism of the oral-gut axis in health and disease. **(A)**: Oral Symbiosis and Homeostasis. Under physiological conditions, a eubiotic oral microbiota supports intact bone integrity and tissue homeostasis, maintaining a compartmentalized axis with limited microbial translocation to preserve systemic health. **(B)**: Gut Symbiosis and Immunity. Simultaneously, a healthy gut microbiome, dominated by commensal bacteria such as Lachnospiraceae, produces essential metabolites, including short-chain fatty acids (SCFAs), that reinforce the epithelial barrier and regulate systemic immunity to actively support oral tissues. **(C)**: Oral-to-Gut Pathogenicity. In contrast, periodontitis instigates a forward mechanism of disease where dysbiotic oral pathogens and toxins translocate via oro-digestive or haematogenous routes, contributing to systemic pathologies such as cardiovascular disease and colorectal carcinoma. **(D)**: Gut-to-Oral Dysregulation. Crucially, the axis functions bidirectionally; gut dysbiosis—characterized by the expansion of pro-inflammatory taxa and depletion of butyrate producers—compromises intestinal barrier function. This “leaky gut” facilitates the systemic influx of lipopolysaccharides (LPS) and inflammatory mediators, which actively exacerbate oral pathologies, including periodontitis and autoimmune disorders, establishing gut microbial imbalance as a driver of oral disease.

For decades, the oral-gut axis has been primarily framed as a forward mechanism: oral pathogens translocate into the gastrointestinal tract, where they contribute to systemic inflammation, metabolic disturbances, and gut pathologies, such as inflammatory bowel disease (IBD) and colorectal cancer (Lam et al., 2023). This concept has shaped both research and clinical practice, emphasizing oral health as a determinant of systemic health. However, the evidence synthesized in this review warrants a reorientation of this narrative. Emerging evidence suggests that gut microbial dysbiosis may influence oral health outcomes, not merely as a downstream response to oral pathogens but through systemic immune modulation, metabolic signaling, and microbial community interactions documented in recent mechanistic and causal inference studies (Baima et al., 2024; Kawamoto et al., 2021). As mentioned above, recognizing this bidirectional relationship is important for advancing microbiome research and for refining clinical perspectives in dentistry, as it encourages a more integrated view of oral health within broader host–microbiome interactions and systemic disease processes (Figure 1).

The most substantial evidence for gut-to-oral influence emerges in autoimmune and chronic inflammatory diseases with oral manifestations. Primary Sjögren's syndrome (pSS), characterized by salivary gland destruction and xerostomia, has consistently been linked to gut microbial alterations (Li et al., 2022; Mendez et al., 2020). Patients with pSS exhibit reduced microbial diversity and enrichment of pro-inflammatory taxa such as *Escherichia*–*Shigella* (Yang et al., 2022). Mendelian Randomization (MR) studies provide causal evidence, identifying protective roles of different genera, specifically *Eubacterium coprostanoligenes*, modulating systemic inflammatory markers through CXCL6 levels (Wang et al., 2024). These findings suggest that gut dysbiosis may prime systemic autoimmunity, which then manifests in oral tissues. Similarly, recurrent aphthous ulcers (RAU) and Behçet's disease (BD) feature in the gut's systemic reach. In BD, disease activity correlates with shifts in the fecal microbiome (Kim et al., 2021), whereas RAU has been linked to small intestinal bacterial overgrowth (Liu et al., 2025) and specific gut taxa *via* MR analyses (Jin et al., 2024). Collectively, these studies highlight the gut microbiome's capacity to drive oral autoimmunity through systemic immune dysregulation.

Gut-derived metabolites, beyond their role in immune modulation, act as key mediators influencing oral health, affecting processes such as inflammation, tissue integrity, and microbial balance. Short-chain fatty acids (SCFAs), particularly butyrate, play a crucial role in regulating inflammation and epithelial integrity by inhibiting histone deacetylases and reinforcing tight junction integrity, respectively (Li et al., 2021; Xu et al., 2024). Individuals with periodontitis consistently show a decreased presence of butyrate-producing bacteria, particularly those belonging to the *Lachnospiraceae* family (Ye et al., 2023; Lin et al., 2025). This systemic SCFA deficit may compromise oral mucosal immunity, exacerbating chronic gingival inflammation. The analogy between periodontitis and IBD, both characterized by impaired barrier function and dysregulated immune responses, further supports the concept of a shared immunopathogenesis (Zilberstein et al., 2025; Amado et al., 2020). In dental caries, alterations in gut microbial diversity may influence nutrient metabolism and immune signaling (Wang et al., 2025), indirectly shaping the composition and ecological balance of the oral plaque microbiome (Zheng et al., 2023). These findings suggest that metabolite signaling is perhaps a key mechanistic bridge between gut dysbiosis and oral disease.

The gut's influence extends to acute oral conditions, such as mucositis, and to malignancies, such as oral squamous cell carcinoma (OSCC). In mucositis, chemotherapy-induced gut dysbiosis is characterized by an increase in Bacteroidetes and a decrease in Proteobacteria (Chen et al., 2024), and correlates with more severe oral mucosal injury (Bartha et al., 2025). This suggests that gut barrier disruption permits systemic inflammatory signals to exacerbate local oral damage. In OSCC, distinct gut and oral microbial signatures have been identified, with taxa such as *Porphyromonas* and *Prevotella* implicated in tumor progression and treatment response (Matsui et al., 2024). Additionally, the presence of *Clostridium Subcluster XIVa* in the gut flora increases PD-L1 expression, thereby modulating the tumor immune response. These findings suggest that oral and gut microbiota signatures serve as putative biomarkers for oral cancer risk and prognosis, thereby opening new avenues for microbiome-based oncology.

Interestingly, a preliminary study in healthy older individuals with intact dentition showed minimal microbial overlap between oral and gut sites (Nishimoto et al., 2023). This suggests that under physiological conditions, the oral-gut axis is relatively compartmentalized, with limited microbial translocation. Disruptions to this barrier appear to emerge predominantly in pathological states characterized by microbial dysbiosis. Such observations reinforce the hypothesis that disease-associated crosstalk between the gut and oral microbiomes is not constitutive but condition-dependent, thereby demanding further mechanistic investigation. Importantly, this supports the emerging concept that gut microbial imbalance may also act as a driver of oral disease progression rather than merely a passive correlate or downstream consequence.

## Limitations of the current gut–oral axis research

Although there are associations, several limitations restrain the current perspectives of the oral-gut microbiome relationship. Most studies are observational, which restricts causal inference. MR analyses offer stronger support but are limited by the availability of genetic instruments and population differences. The mechanistic pathways, especially the specific roles of metabolites and immune mediators, are not fully understood. Additionally, few interventional studies have tested microbiota-targeted therapies for oral conditions. Overcoming these gaps requires collaboration among experts in dentistry, gastroenterology, immunology, and microbiome science.

## Clinical implications and future perspectives

The recognition of gut-to-oral influence carries deep clinical implications. Initially, it calls for integrated diagnostics that assess both oral and gut microbiota. Profiling gut microbial composition may aid in the early detection of oral diseases with systemic components, such as pSS or periodontitis. Further, it opens the door to microbiota-targeted therapies. Probiotics, prebiotics, and fecal microbiota transplantation (FMT) could complement conventional oral treatments, particularly in chronic inflammatory conditions. Furthermore, it emphasizes the importance of personalized medicine. By integrating gut-oral microbiome data, clinicians may tailor interventions to individual microbial profiles, improving outcomes in autoimmune and inflammatory oral diseases.

To advance this field, future research should focus on mechanistic studies that link specific gut bacteria and metabolites to oral disease outcomes. This effort should include metabolomics profiling to measure SCFAs, bile acids, and Trimethylamine N-oxide (TMAO) precursors in serum and saliva. The effects of probiotics or FMT in patients with pSS, RAU, or periodontitis could be assessed in controlled clinical trials, immunological studies could be conducted to understand how gut-derived cytokines and immune cells affect oral tissues, and systems biology approaches could integrate multi-omics data to map the oral–gut axis at a molecular level. Consequently, field exploration will help clarify how the gut shapes oral health and could lead to innovative therapies that bridge dental care with broader systemic health.

## Data Availability

The original contributions presented in the study are included in the article/supplementary material, further inquiries can be directed to the corresponding author.
